# The Subcutaneous Injection of Paraffin in the Treatment of Saddle Nose

**Published:** 1902-05-10

**Authors:** 


### THE SUBCUTANEOUS INJECTION OF PARAFFIN IN THE TREATMENT OF SADDLE NOSE.

We have several times referred to the treatment of deformities due to loss or depression of the bones of the nose by the subcutaneous injection of melted paraffin, a proceeding which was we believe originated in Vienna. This method has now been applied in a fair number of cases in this country and with considerable success. A case of this sort was lately reported by Dr. Scanes Spicer.[1] The patient, aged 25, had a well marked tip-tilted saddle nose and stunting of the nasal framework. She had suffered from childhood from nasal suppuration and fœtor. For the injection a 15-minim German glass hypodermic syringe was used, like that used for injecting tuberculin. Ten or twelve syringefuls of paraffin were injected under the skin, some downwards over the nasal bones, some upwards from the sides of the nose into the depressed gap, and the injected paraffin was moulded by an assistant's finger so as to shape the parts before setting. The points of injection were sealed up with collodion. The result so far as the appearance of the nose goes, is a very palpable bolstering up of the skin over the bony bridge of the nose and the formation of a very decent sized organ. The operation was performed ten months ago, and as the paraffin remains in the same state as when first injected, it may be regarded, says Dr. Spicer, as permanent, so far as we can see at present. Unfortunately in this case a small nodule of paraffin passed into the upper eyelid, from which it has not been possible to remove it, and there has been some œdema of the eyelids possibly from blockage of the lymphatic vessels by the paraffin. Dr. Downie Walker[2] has also recorded a couple of cases in which this treatment was adopted with great success. The amount of paraffin injected was in one case a little over two drachms, and in the other "fully one drachm." While solidifying it was quickly moulded into the desired shape. "Sterilised paraffin," with a melting point of 104° Fahr. was the material used, and the nose was prepared as for any other surgical operation. The paraffin in a molten condition was injected by means of a serum syringe, the nose being kept warm by hot dry sponge cloths, and the needle kept hot by an electric current. Pressure was applied around the nose and at the inner canthus of the eyes to confine the fluid to the nasal region.

[1] Clinical Jour., April 9. [2] Brit. Med. Jour., May 3.

